# *Eimeria acervulina* Microneme Protein 3 Inhibits Apoptosis of the Chicken Duodenal Epithelial Cell by Targeting the Casitas B-Lineage Lymphoma Protein

**DOI:** 10.3389/fvets.2021.636809

**Published:** 2021-05-18

**Authors:** Pu Wang, Yukun Jia, Yue Han, Weirong Wang, Yiran Zhu, Jiali Xu, Chiyu Guan, Jinpeng Ying, Simin Deng, Jing Wang, Xian Zhang, Mianmian Chen, Changyong Cheng, Houhui Song

**Affiliations:** ^1^Key Laboratory of Applied Technology on Green-Eco Healthy Animal Husbandry of Zhejiang Province, Zhejiang Provincial Engineering Laboratory for Animal Health Inspection and Internet Technology, College of Animal Science and Technology, College of Veterinary Medicine, Zhejiang A&F University, Hangzhou, China; ^2^Key Laboratory of Zoonosis Research, Ministry of Education, College of Veterinary Medicine, Jilin University, Changchun, China; ^3^Jixian Honors College, Zhejiang A&F University, Hangzhou, China

**Keywords:** flow cytometry, caspase-3, MIC3, *Eimeria acervulina*, duodenal epithelial cells

## Abstract

*Eimeria acervulina* (*E. acervulina*) causes coccidiosis in poultry which persists as economic pain worldwide. Most damage to the intestinal mucosa results from apoptosis of the infected intestinal epithelial cells. The Microneme protein 3 (MIC3) protein is a key virulence factor in some parasites involved in host cell apoptosis inhibition. Here, we studied whether and how MIC3 affects the apoptosis in *E. acervulina* infected chicken duodenal epithelial cells. Through flow cytometry (FCM), we found that the presence of merozoites and the overexpression of MIC3 significantly decreased apoptosis and the activity of caspase-3 in chicken duodenal epithelial cells at 4, 6, and 8 h post merozoite infection (*P* < 0.01). Silencing the Casitas B-lineage lymphoma (CBL) protein, a host receptor for MIC3 with shRNA was shown to promote apoptosis in the chicken duodenal epithelial cells. The early apoptotic rate of host cells in the lentiviral-MIC3 group was significantly lower than that in the lentiviral-MIC3 + shRNA CBL group at 4 h after MIC3 expression (*P* < 0.01), and it was moderately decreased in the lentiviral-MIC3 + shRNA CBL group compared with that in the shRNA CBL group. Our data indicated that MIC3 inhibited early apoptosis of *E. acervulina* infected chicken duodenal epithelial cells by targeting host receptor-CBL protein. These findings unveiled one of the mechanisms of how intracellular parasites affect the apoptosis of infected host cells, which provided a deeper understanding of their pathogenesis.

## Introduction

*Eimeria acervulina* (*E. acervulina*) can cause pronounced pathological intestinal changes and death in infected chickens (1). Chickens are infected by ingesting *E. acervulina*-sporulated oocysts contaminated feed. Sporozoites are released from the oocysts by gastric acid digestion and duodenal peristalsis. Each sporozoite undergoes multiple rounds of division to form multiple merozoites, which cause cell damage (2). It has been reported that damage to *E. acervulina* infected host cells is mainly caused by apoptosis (3).

Apoptosis represents a physiological form of cell death that occurs in most multicellular organisms. It is one of the most important defensive measures employed by hosts to resist or eliminate pathogen infection (4). Some parasites are known to delay host cell apoptosis to promote their growth and reproduction in the early stage of parasite infection (5–7). It has been confirmed that intracellular parasites such as *Cryptosporidium parvum (C. parvum), Trypanosoma cruzi (T. cruzi), Theileria parvum (T. parvum), Toxoplasma gondii (T. gondii), Neospora caninum (N. caninum), Leishmania, and Plasmodium* can inhibit host cell apoptosis after invading cells (8–14). For example, *T. gondii* can inhibit the apoptosis of host T cells and macrophages, thus prolonging the survival time of infected cells to maintain their continued replication in host cells (8). *T. cruzi* can inhibit cell apoptosis mediated by Fas and TNF-α (15). *T. parvum, C. parvum*, and *N. caninum* use the NF-κB activation pathway to inhibit the cell apoptosis to enable their continued replication in host cells or their escape from the immune attack of host cells (11, 12, 16). *Eimeria* is also an intracellular parasite, and it has been found that *Eimeria* can inhibit host cell apoptosis in the early stage of infection (13). The second-generation schizonts of *Eimeria necatrix* have been shown to inhibit host cell apoptosis by activating the NF-κB signaling pathway in the early stage of infection (16). *Eimeria bovis* sporozoites can inhibit the host cell apoptosis by increasing the expression of the anti-apoptotic factors c-IAP and c-FLIP (17, 18). After *E. acervulina* merozoites invaded bovine kidney cells, cell apoptosis was inhibited in the early stage (19). However, it has not been reported how *E. acervulina* may affect the apoptosis in duodenal epithelial host cells.

Micronemes are the smallest secretory organelles in the apices of apicomplexan parasites and are mainly found in sliding and invading parasites (20). Microneme protein 2 (MIC2), MIC3, and MIC5 have been found in *E. acervulina*, and MIC3 is the most protective functional antigen in the parasite (21). MIC3 mediates sporozoite invasion and determines the specificity of the invasion site (22). This protein is a key virulence factor in parasites that can inhibit host cell apoptosis. The functional domains of epidermal growth factor (EGF) in MIC3 and MIC6 of *T. gondii* activate the EGFR-AKT signaling pathway in host cells, inhibiting the killing effects of the host cells and promoting parasite survival (15). However, *E. acervulina* MIC3 has no functional EGF domain and cannot bind to the EGFR receptor in chicken duodenal epithelial cells. Whether the molecular mechanism involves specific receptors other than EGFR in duodenal epithelial cells remains elusive. In previous studies, the interaction between *E. acervulina* MIC3 and its receptor, CBL (Casitas B-lineage lymphoma), was identified in duodenal epithelial host cells (23). The CBL protein family belongs to E3 ubiquitin ligases, including c-Cbl, Cbl-b, and Cbl-c. They function as E3 ligases and regulators of apoptosis signal transduction (24, 25).

In this study, Annexin V-FITC/PI and TUNEL assays were used to detect the effects of *E. acervulina* and MIC3 on the apoptosis in duodenal epithelial cells. Furthermore, an shRNA targeting the CBL protein was designed and synthesized to silence the gene encoding CBL protein. Increased apoptosis of chicken duodenal epithelial cells was detected with downregulated expression of CBL protein. Finally, we studied the interplay of MIC3 protein and CBL in the apoptosis of chicken duodenal epithelial cells. The results helped reveal the pathogenic mechanism of *E. acervulina* infecting the chicken duodenal epithelial host cells.

## Materials and Methods

### Parasites

A Guangdong strain of *E. acervulina* maintained in our laboratory was used in this study. The oocysts used in the study were freshly prepared (≤ 1 month of storage). Separation and purification of the merozoites were carried out by DE-52 chromatography.

### Primary Culture of Chicken Duodenal Epithelial Cells

Chicken duodenal epithelial cells were collected from 18-day-old SPF chick embryos (Merial Vital Corp, Beijing, China). Briefly, cells were digested with 50 mg/L thermolysin (Sigma, St. Louis, MO, USA) at 41°C for 2 h, and dissociated cells were immediately placed in DMEM supplemented with 10 % fetal bovine serum (FBS; Sijiqing Corp., Hangzhou, China), 100 U/mL penicillin, 100 U/mL streptomycin, 0.02 μg/mL EGF, 0.1 mg/mL heparin, 1.1 mg/mL sodium pyruvate, 0.05 μg/mL insulin and 0.5 mM L-glutamine. Suspensions were incubated at 41°C for 70 min in a humidified incubator with 8% CO_2_ to allow some cells to adhere and separate. Non-adherent cell aggregates were resuspended for use in subsequent experiments using DMEM supplemented with 2.5% FBS and the other factors described above. The study was approved by the animal ethics committee of the College of Animal Science and Technology of Zhejiang A&F University.

### MTT Assays

The viability of chicken duodenal epithelial cells exposed to actinomycin D (Act D; Meilun Corp., Dalian, China) was evaluated using 3-(4,5-dimethylthiazol-2-yl)-2,5-diphenyltetrazolium bromide (MTT) assays. Chicken duodenal epithelial cells were plated in 96-well plates at a density of 1.0 × 10^4^ cells/well and grown for 48 h. The culture medium was then replaced with fresh medium supplemented with 5, 10, 20, or 40 μM Act D, and the cultures were incubated for another 4 h. Cells were then treated for 4 h at 41°C with sterile-filtered MTT reagent (Solarbio, Beijing, China). The culture supernatants were removed by aspiration, and the formazan crystals present were dissolved in DMSO (Solarbio Beijing, China). The absorbance was measured at 492 nm.

### The Effect of *E. acervulina* Merozoites on Chicken Duodenal Epithelial Cell Apoptosis

In the next experiment, we isolated and cultured chicken duodenal epithelial cells, purified the merozoites, and established a duodenal epithelial cell apoptosis model using Act D as the apoptosis inducer. Flow cytometry with Annexin V-FITC/PI double staining was used to detect apoptosis. The effects of Act D on chicken duodenal epithelial cell apoptosis and viability were analyzed. After the cells reached 90% confluence, they were divided into the following four groups: a blank control group (A0), a merozoite group (A2), an Act D group (A3), and a merozoite + Act D group (A4). Act D was added to the appropriate cells at 0, 2, or 4 h after infection with 10^5^ *E. acervulina* merozoites. The cells were harvested after 4 h of drug treatment. Apoptosis was detected with an Annexin V-FITC/PI apoptosis kit (Bio Vision, Milpitas, CA, USA). Caspase-3 activity was analyzed using a Caspase-3 Activity Assay Kit (Beyotime, Shanghai, China).

### Construction and Detection of a Recombinant Lentivirus Carrying MIC3 Protein

The complete sequence of the MIC3 gene (GenBank Accession No. KU359773), which encodes seven microneme adhesive repeat (MAR) domains, was cloned into the pLVX-IRES-Puro-Flag vector. The vector carrying genes of MIC3 and green fluorescent protein was then constructed and transfected into 293T cells. The recombinant lentivirus was harvested after 72 h. Chicken duodenal epithelial cells were then infected with the recombinant lentivirus. The infection efficiency of the lentivirus was examined by immunofluorescence to determine the multiplicity of infection (MOI) of the lentivirus.

### The Effect of MIC3 Protein on the Apoptosis of Chicken Duodenal Epithelial Cell

A lentivirus vector containing the gene of MIC3 alone was constructed, and the expression of MIC3 was detected at different time points in chicken duodenal epithelial cells *via* western blotting. Early and late apoptosis in chicken duodenal epithelial cells was detected after infection with lentivirus containing MIC3 but not green fluorescent protein (A0 group). Lentiviral vector blank group (A2), The negative control groups were an Act D group (A3) and a Lentiviral - MIC3 + Act D group (A4). Act D was added to the appropriate cells at 0, 2, or 4 h after MIC3 expression. The cells were harvested after 4 h of drug treatment. Apoptosis was detected with an Annexin V-FITC/PI apoptosis kit. Caspase-3 activity was analyzed with a Caspase-3 Activity Assay Kit.

### The Effect of shRNA Targeting CBL Protein on Chicken Duodenal Epithelial Cell Apoptosis

shRNAs targeting the CBL protein were designed and synthesized by OBIO Technology Corp., Ltd. (Shanghai, China). The shRNAs were cloned into the pLKD-CMV-Puro-U6-EGFP vector, and the CBL gene was cloned into the pLVX-IRES-Puro-Flag- EGFP vector. The pLKD-CMV-Puro-U6-EGFP-shRNA and pLVX-IRES-Puro-Flag-EGFP-CBL plasmids were transfected into 293T cells. The recombinant lentivirus was harvested after 72 h. Chicken duodenal epithelial cells were then infected with recombinant lentivirus carrying the shRNA and CBL genes. CBL expression was detected by Western blot and RT-PCR. To detect the effect of CBL on apoptosis, the pLKD-CMV-Puro-U6 vector carrying the shRNA and the pLVX-IRES-Puro-Flag vector carrying CBL were constructed and transfected into 293T cells. The recombinant lentivirus was harvested after 72 h. Chicken duodenal epithelial cells were then infected with recombinant lentivirus carrying the shRNA and CBL genes. The cells were harvested after infection for 72 h.

### The Effect of MIC3 Protein on Chicken Duodenal Epithelial Cell Apoptosis Through Targeting CBL

Previous research has shown that the MIC3 protein interacts with the CBL protein intracellularly (23). To explore the relationship between the inhibitory effect of MIC3 on apoptosis and the function of CBL, apoptosis was detected in chicken duodenal epithelial cells by TUNEL assay. Chicken duodenal epithelial cells were infected with recombinant lentivirus containing MIC3 protein (MOI of 40) after 72 h and then infected with recombinant lentivirus carrying the shRNA. Apoptosis was detected with a TUNEL apoptosis assay kit (Beyotime, Shanghai, China) and an Annexin V-FITC/PI apoptosis kit.

## Results

### Cell Viability

The effect of Act D on chicken duodenal epithelial cell viability was investigated using MTT and Annexin V-FITC/PI assays. The Act D concentration was selected to promote a cell death rate of no more than 50% and an apoptosis rate of 30% as the formal test conditions. The MTT and flow cytometry assays results showed that the apoptosis rate of duodenal epithelial cells was ~30%, and the death rate was <50% when the cells were treated with 20 μg/mL Act D for 4 h (Tables 1, 2).

**Table 1:** Effect of Act D on the hostcell survival rate.

**Drug concentration**	**Host cell activity (OD570 nm)**	**Survival rate (%)**
—	0.258 ± 0.02113	100
0.5%DMSO	0.245 ± 0.002657	—
5 μg/mL	0.199 ± 0.002573	71.25
10 μg/mL	0.184 ± 0.002682	67.53
20 μg/mL	0.174 ± 0.00208	60.83
40 μg/mL	0.167 ± 0.00178	53.57

**Table 2:** Effect of Act D on the host cell apoptosis rate.

**Experimental groups**	**Early apoptosis rate of host cells (%)**	**Late apoptosis rate of host cells (%)**	**Total apoptosis rate of host cells (%)**
—	5.59 ± 0.34	3.55 ± 0.37	9.14 ± 0.71
10 μg/mL	7.63 ± 0.17	17.56 ± 0.26	25.19 ± 0.43
20 μg/mL	12.37 ± 1.18	29.64 ± 1.34	42.01 ± 2.52
40 μg/mL	17.87 ± 1.43	34.75 ± 1.06	52.62 ± 2.49

### The Effect of *E. acervulina* Merozoites on Chicken Duodenal Epithelial Cell Apoptosis

**Figure 1:**
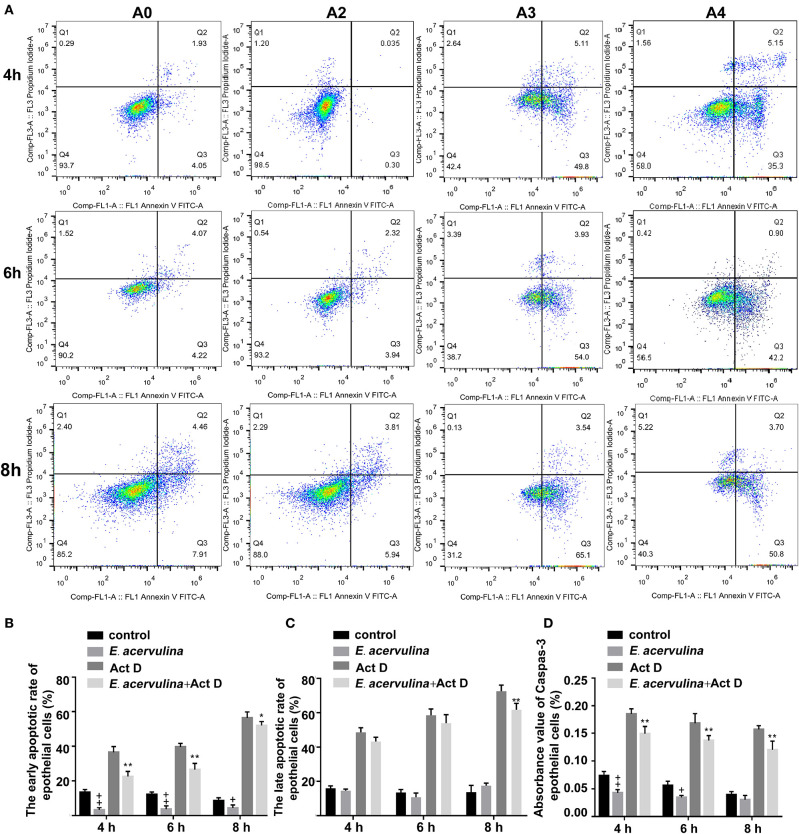
Effects of early and late apoptosis on epithelial host cells after *E. acervulina* infection. **(A)** The apoptosis/necrosis rate of chick duodenal epithelial cells was detected by Annexin V/PI assay after *E. acervulina* merozoite infection. **(B)** Quantitative determination of the early apoptosis rate (*n* = 3). ^+^*P* < 0.01 vs. the control. **(C)** Quantitative determination of the late apoptosis/necrosis rate (*n* = 3). ^+^*P* < 0.01 vs. the control. **(D)** Analysis of caspase-3 activity after *E. acervulina* merozoite infection. ^+^*P* < 0.01 vs. the control. “+” represents the apoptotic rate of host cells in *E. acervulina* merozoite group was significantly lower than that in the blank control group (^+^*P* < 0.05). “$\underset{+}{+}$” represents the apoptotic rate of host cells in *E. acervulina* merozoite group was significantly lower than that in the blank control group (^+^*P* < 0.01). “_*_” represents the early apoptotic rate of host cells in *E. acervulina* merozoite + Act D group was significantly lower than that in the Act D group (^+^*P* < 0.05). “_**_” represents the early apoptotic rate of host cells in *E. acervulina* merozoite + Act D group was significantly lower than that in the Act D group (^+^*P* < 0.01). *T*-test were used to statistically analyze the data. A0 represents the blank control group. A2 represents *E. acervulina* infection group. A3 represents Act D infection group. A4 represents *E. acervulina* + Act D infection group.

The apoptosis/necrosis rate of chicken duodenal epithelial cells was detected by Annexin V/PI assay after *E. acervulina* merozoite infection (Figure 1A). The early apoptotic rate of the host cells in the merozoite group was significantly lower than that in the blank control group at 4 h after merozoite infection (*P* < 0.01). The early apoptotic rate of the host cells in the merozoite + Act D group was significantly lower than that in the Act D group (*P* < 0.01) at 4–8 h, indicating that *E. acervulina* infection resulted in the inhibition of Act D-induced host cell apoptosis (Figure 1B). The late apoptotic/necrosis rate of the host cells in the merozoite group was lower than that in the blank control group at 4–8 h after merozoite infection. However, the difference was not significant (Figure 1C). Caspase-3 activity (as measured by the absorbance 405 nm value) in the merozoite group was significantly lower than that in the blank control group (*P* < 0.01) at 4 h after merozoite infection. Additionally, caspase-3 activity in the merozoite group was lower than that in the blank control group at 8 h after merozoite infection. Caspase-3 activity in the merozoite + Act D group was significantly lower than that in the Act D group (*P* < 0.01) at 4, 6, and 8 h after infection (Figure 1D). In this study, we examined the mechanisms mediating *E. acervulina*-inhibited apoptosis in chicken duodenal epithelial cells. Our data indicated that the inhibition of cell apoptosis may have a relationship with the triggering of caspase-3.

### Construction and Detection of a Recombinant Lentivirus Carrying MIC3 Protein

**Figure 2:**
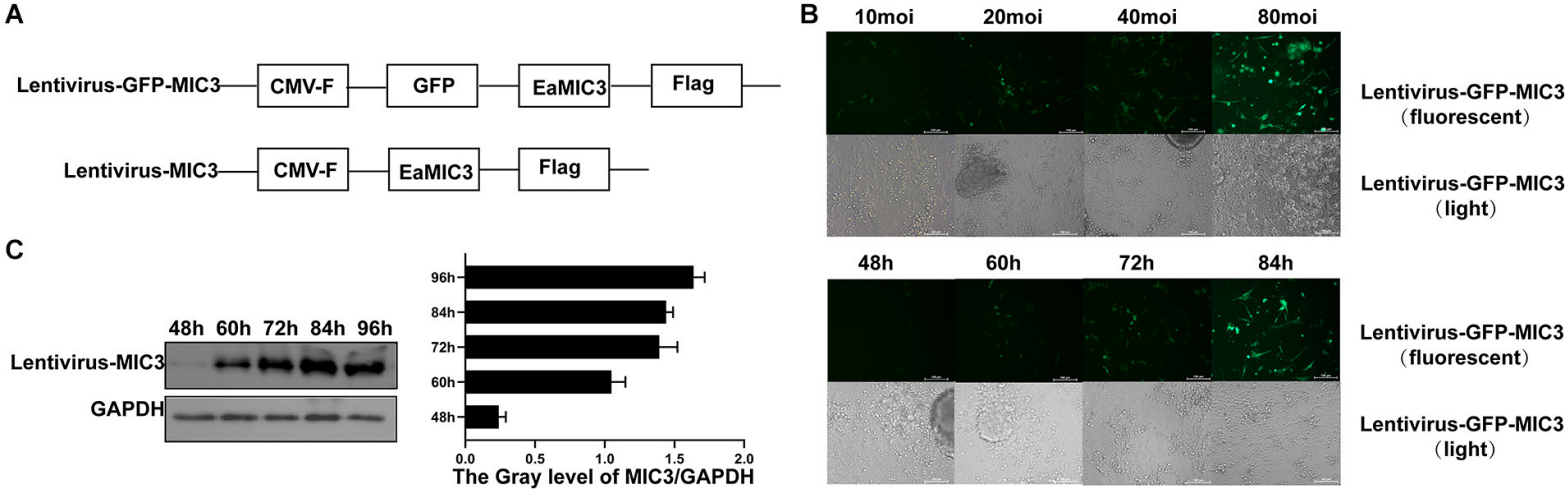
Construction and detection of a recombinant lentivirus carrying MIC3 protein. **(A)** The construction strategy for the lentiviral vector carrying MIC3/green fluorescent protein or the MIC3 protein alone. **(B)** The cells were infected using recombinant lentivirus carrying MIC3/green fluorescent protein, and the optimal multiplication of infection (MOI) was obtained through the detection of green fluorescent protein expression by flow cytometry. **(C)** MIC3 was expressed in chick duodenal epithelial cells at different times. Western blot and gray analyses were used to study MIC3 expression in chick duodenal epithelial cells. The apoptosis/necrosis rate of chick duodenal epithelial cells was detected by Annexin V/PI assay after MIC3 expression.

Lentivirus vectors carrying the MIC3 and green fluorescent protein gene or the MIC3 gene alone were successfully constructed (Figure 2A). MIC3 and green fluorescent protein were successfully expressed in duodenal epithelial cells. The optimal MOI was 40 at 72 h after infection with lentivirus carrying the MIC3 and green fluorescent proteins (Figure 2B). MIC3 protein expression began at 48 h and increased with time after infection with recombinant lentivirus carrying MIC3 (Figure 2C).

### The Effect of MIC3 Protein on Chicken Duodenal Epithelial Cell Apoptosis

**Figure 3:**
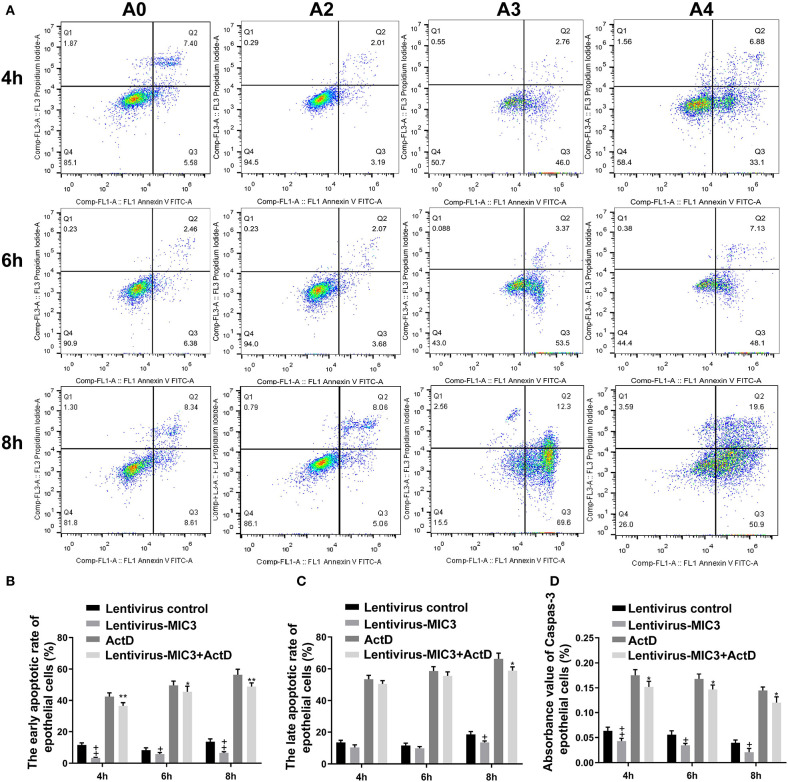
Effects of early and late apoptosis on epithelial host cells after MIC3 protein expression. **(A)** The apoptosis/necrosis rate of chick duodenal epithelial cells was detected by Annexin V/PI assay after MIC3 expression. **(B)** Quantitative determination of the early apoptosis/necrosis rates after MIC3 expression (*n* = 3). **(C)** Quantitative determination of the late apoptosis/necrosis rates after MIC3 expression (*n* = 3). **(D)** Caspase-3 activity after MIC3 expression (*n* = 3). ^+^*P* < 0.01 vs. the control. “^+^” represents the apoptotic rate of host cells in Lentivirus-MIC3 group was significantly lower than that in Lentivirus control group (^+^*P* < 0.05). “$\underset{+}{+}$” represents the apoptotic rate of Lentivirus-MIC3 group was significantly lower than that in the Lentivirus control group (^+^*P* < 0.01). “*” represents the early apoptotic rate of host cells in Lentivirus-MIC3 + Act D group was significantly lower than that in Act D group (^+^*P* < 0.05). “**” represents the early apoptotic rate of Lentivirus-MIC3 + Act D group was significantly lower than that in the Act D group (^+^*P* < 0.01). *T*-test were used to statistically analyze the data. A0 represents the blank control group. A2 represents MIC3 protein infection group. A3 represents Act D infection group. A4 represents MIC3 + Act D infection group.

The apoptosis/necrosis rate of chicken duodenal epithelial cells was detected by Annexin V/PI assay after MIC3 protein expression (Figure 3A). The early apoptotic rate of host cells in the Lentiviral - MIC3 group was significantly lower than that in the Lentiviral blank control group and the Lentiviral - MIC3 + Act D group at 4-8 h after MIC3 expression (*P* < 0.01) (Figure 3B). This result suggests that *E. acervulina* MIC3 inhibits duodenal epithelial cell apoptosis in the early stage and that it can inhibit duodenal cell apoptosis induced by Act D. The late apoptotic/necrosis rate of host cells in the Lentiviral - MIC3 group was lower than that in the blank control group at 4–8 h after MIC3 expression. However, the difference was not significant (Figure 3C). Notably, the late apoptotic/necrosis rate of host cells in the Lentiviral - MIC3 group was significantly lower than that in the blank control group at 8 h after MIC3 expression (*P* < 0.05). Caspase-3 activity (measured as absorbance 405 nm value) in the Lentiviral - MIC3 group was significantly lower than that in the blank control group (*P* < 0.05) at 4–8 h after MIC3 expression (Figure 3D).

### The Effect of shRNA Targeting CBL Protein on the Apoptosis of Chicken Duodenal Epithelial Cell

**Figure 4:**
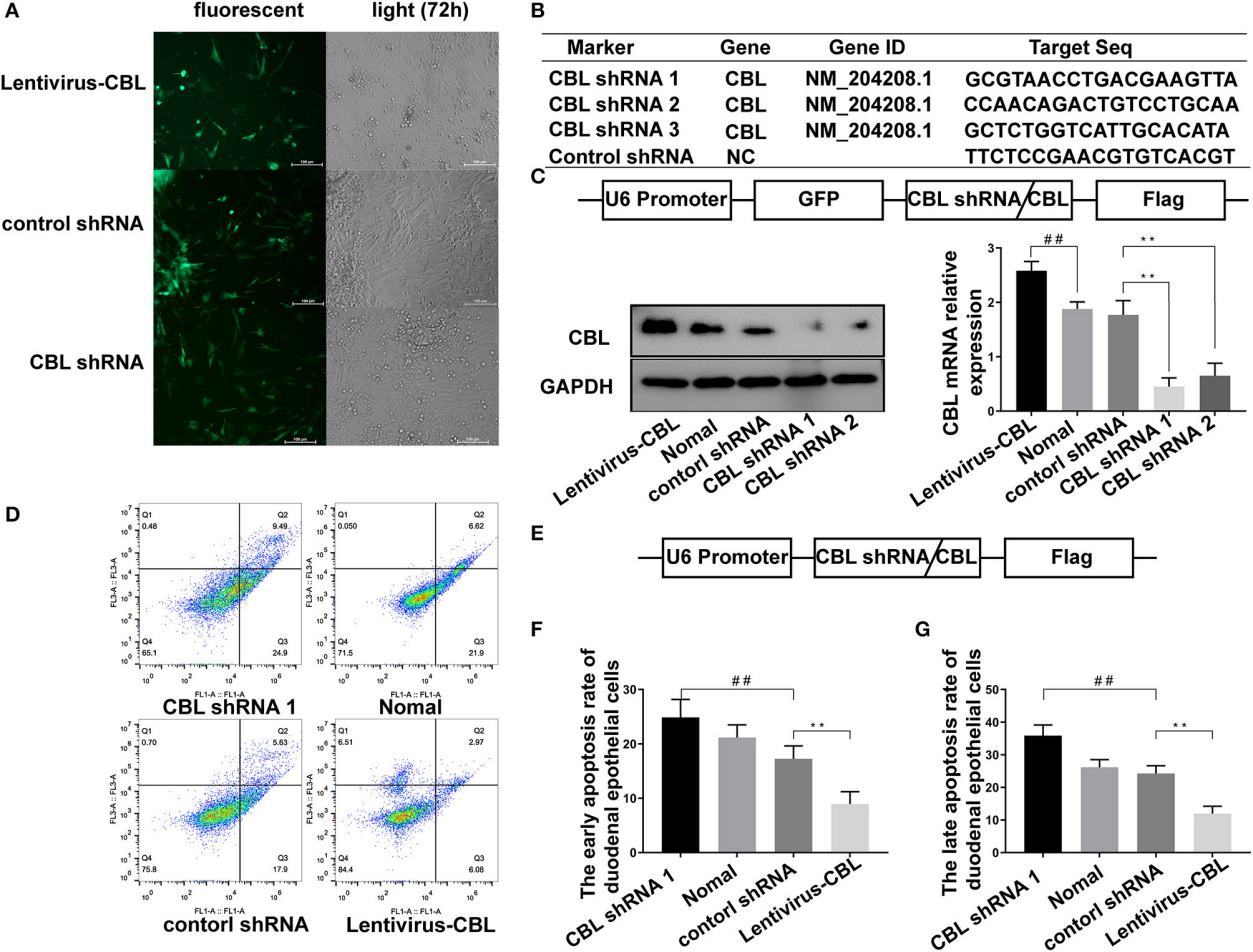
The effect of shRNA targeting CBL protein on chick duodenal epithelial cell apoptosis. **(A)** CBL shRNA and green fluorescent protein were successfully expressed in duodenal epithelial cells. **(B)** Three shRNAs targeting the CBL mRNA and one blank shRNA were designed and synthesized. **(C)** The construction strategy for the CBL shRNA and overexpression vector is displayed. Western blot and RT-PCR results show CBL protein expression in the CBL shRNA, normal and lentivirus-CBL groups at 72 h after infection. **(D)** Flow cytometry shows the apoptosis rate of chick duodenal epithelial cells in the control, CBL shRNA, lentivirus-CBL, and normal groups. **(E)** The construction strategy for the lentivirus vectors carrying CBL shRNA/CBL without green fluorescent protein. “_##_” represents the apoptotic rate of host cells in control shRNA group was significantly lower than that in the CBL shRNA group (^+^*P* < 0.01). **(F)** Quantitative analysis of the early apoptosis rate of chick duodenal epithelial cells in the control, CBL shRNA, lentivirus-CBL, and normal groups. **(G)** Quantitative analysis of the late apoptosis rate of chick duodenal epithelial cells in control, CBL shRNA, lentivirus-CBL, and normal groups. “_**_” represents the apoptotic rate of host cells in Lentivirus-CBL group was significantly lower than that in the control shRNA group (^+^*P* < 0.01).

An shRNA targeting the CBL protein was designed, and lentivirus vectors carrying CBL shRNA were successfully constructed. CBL shRNA and green fluorescent protein were successfully expressed in duodenal epithelial cells (Figure 4A). Three shRNAs targeting the CBL mRNA and one blank shRNA were designed and synthesized (Figure 4B). The CBL shRNA and overexpression vector construction strategy is shown. Compared with the control group, western blot and RT-PCR results showed that CBL shRNA 1 and shRNA 2 significantly inhibited CBL protein expression at 72 h after infection (*P* < 0.01). Lentivirus vectors carrying CBL significantly promoted the expression of CBL protein at 72 h after infection (*P* < 0.01) (Figure 4C). Flow cytometry showed the apoptosis rate of the control, CBL shRNA, the lentivirus-CBL, and normal groups (Figure 4D). To more accurately obtain the apoptosis rate, lentivirus vectors carrying CBL shRNA/CBL without green fluorescent protein were successfully constructed (Figure 4E). The early apoptotic rate of host cells in the CBL shRNA group increased significantly than that in the control shRNA group (*P* < 0.01) (Figure 4F). The late apoptotic/necrosis rate of host cells in the CBL group was significantly lower than that in the control shRNA group (*P* < 0.01) (Figure 4G).

### The Effect of MIC3 Protein on Chicken Duodenal Epithelial Cell Apoptosis Through Targeting CBL

**Figure 5:**
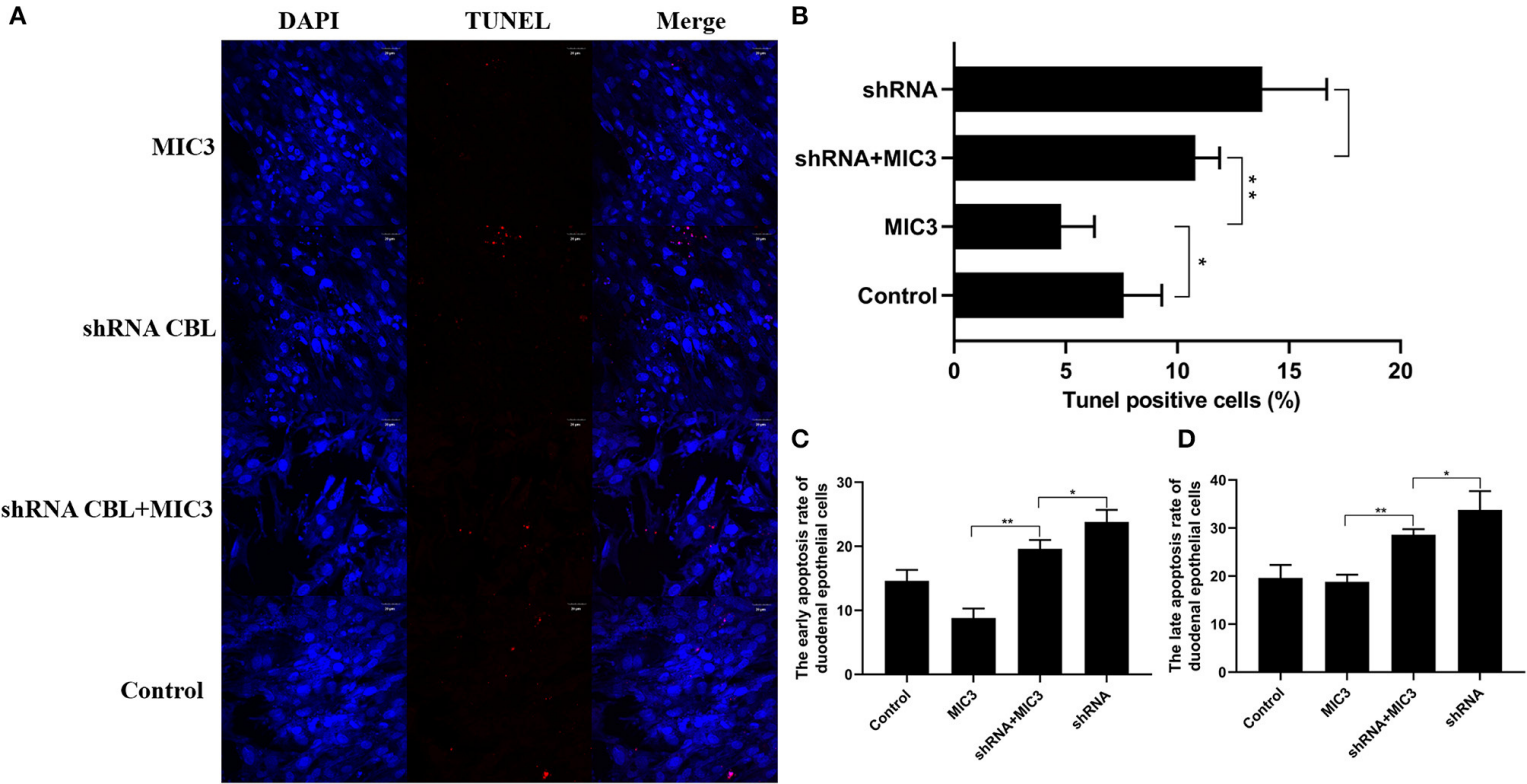
The inhibitory effect of MIC3 protein on the apoptosis of chick duodenal epithelial cells through targeting CBL. **(A)** The observation of emitted red fluorescence under the fluorescence microscope showing the apoptosis rate of chick duodenal epithelial cells in the control, CBL shRNA, CBL shRNA+MIC3, and MIC3 groups by TUNEL assay. **(B)** Quantitative analysis of the TUNEL positive cells in the control group, the CBL shRNA group, CBL shRNA+MIC3, and the MIC3 groups by TUNEL assay (*n* = 3). **(C)** Quantitative analysis of the early apoptosis rate of chick duodenal epithelial cells in the control, CBL shRNA, CBL shRNA+MIC3, and MIC3 groups by Annexin V-FITC/PI apoptosis kit assay (*n* = 3). “_**_” represents the apoptotic rate of host cells in MIC3 group was significantly lower than that in the shRNA+MIC3 group (^+^*P* < 0.01). “_*_” represents the apoptotic rate of host cells in shRNA+MIC3 group was significantly lower than that in shRNA group (^+^*P* < 0.05). **(D)** Quantitative analysis of the late apoptosis rate of chick duodenal epithelial cells in the control, CBL shRNA, CBL shRNA+MIC3, and MIC3 groups by Annexin V-FITC/PI apoptosis kit assay (*n* = 3). ^+^*P* < 0.01 vs. the control. *T*-test were used to statistically analyze the data.

**Figure 6:**
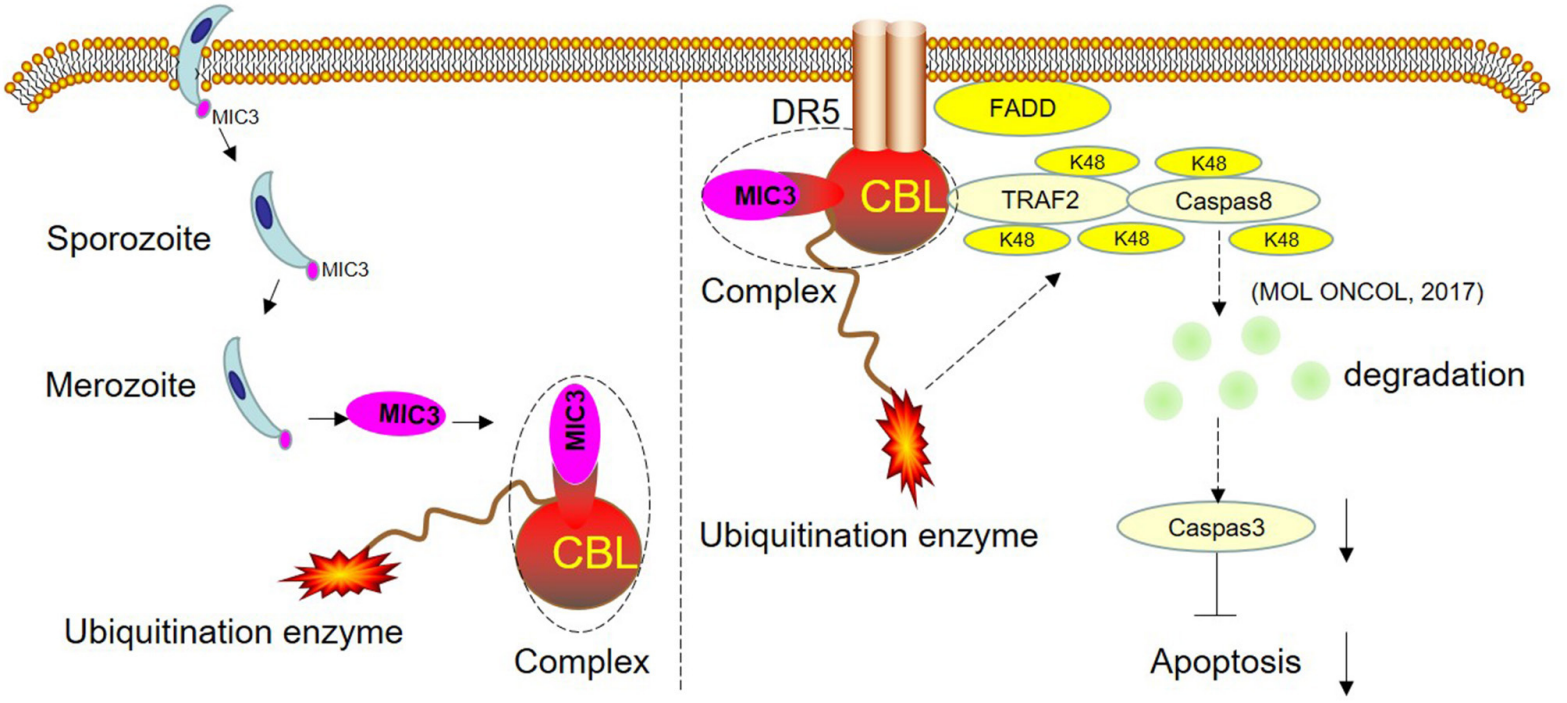
Schematic representation of the proposed model. CBL and EaMIC3 form a complex in chick duodenal epithelial cells. Overexpression of CBL inhibits duodenal epithelial cell apoptosis, while interference of CBL expression promotes apoptosis. CBL are the critical adaptors linking DR5 and TRAF2. TRAF2 mediates the K48-linked polyubiquitination and degradation of caspase-8, which blocks the apoptosis. The shRNA targeting CBL inhibits TRAF2-mediated K48-linked polyubiquitination and degradation of caspase-8, which leads to the induction of apoptosis. The MIC3 inhibits apoptosis of the chick duodenal epithelial cell by targeting CBL.

TUNEL staining is a direct method to visualize the proportion of apoptosis by labeling broken genomic DNA fragments that arise from apoptosis. Stained apoptotic cells emitted red fluorescence under the fluorescence microscope (Figure 5A). The number of TUNEL positive cells in the group with high MIC3 expression was significantly less than that in the control group (*P* < 0.05) through TUNEL assays. The number of TUNEL positive cells in the shRNA CBL+ MIC3 group was not significantly less than that in the shRNA CBL group, but was significantly more than that in the MIC group (*P* < 0.01) (Figure 5B). Annexin V-FITC/PI assays showed that the early apoptotic rate of host cells in the lentiviral-MIC3 group was significantly less than that in the lentiviral-MIC3 + shRNA CBL group 4 h after MIC3 expression (*P* < 0.01). The early apoptotic rate of host cells in the lentiviral-MIC3 + shRNA CBL group was less than that in the shRNA CBL group (*P* < 0.05) (Figure 5C). The late apoptotic/necrosis rate of host cells in the lentiviral-MIC3 + shRNA CBL group was less than that in the shRNA CBL group at 4 h after MIC3 expression (*P* < 0.05). The late apoptotic rate of host cells in the lentiviral-MIC3 group was significantly less than that in the lentiviral-MIC3 + shRNA CBL group at 4 h after MIC3 expression (*P* < 0.01) (Figure 5D). The result of the MIC3 inhibits apoptosis of the chick duodenal epithelial cell by targeting the Casitas B-lineage lymphoma was shown in Figure 6.

## Discussion

During intracellular parasitic infection, host cells participate in a complex network of events that are crucial to the final infection outcomes (26). The modulation of host cell apoptosis by pathogens has attracted the attention of scientists during the last decade. Apoptosis is an efficient mechanism used by the host to control infection and limit pathogen multiplication and dissemination (27). To increase the likelihood of completing their complex life cycles and the transmission between hosts, intracellular parasites have developed mechanisms to block apoptosis and sustain host cell viability. It has been reported that the early apoptotic rate of bovine kidney cells was inhibited by *E. tenella* sporozoites through activation of the PI3K/Akt pathway (28). By applying Annexin V-FITC/PI assays, we confirmed that *E. acervulina* merozoite invasion inhibits host cell apoptosis in the early stage of infection. This finding seems to reflect a general evasion strategy of intracellular apicomplexans and is consistent with data on related parasites, such as other *Eimeria* spp., *T. gondii*, and *N. caninum*. Diverse molecular mechanisms underly the inhibition of apoptosis by different intracellular protozoans. However, the proteins in *E. acervulina* that are involved in the inhibition of host cell apoptosis remain unknown.

Previous studies have shown that microneme proteins (MICs) play important roles in host-cell invasion (29). The majority of MICs are adhesions molecules that bind to host cells during invasion. As one of the most extensively studied MICs, EaMIC3 plays an important role in parasite invasion and colonization. It was reported that EaMIC3 is expressed during the sporozoite and merozoite stages and is located at the apex of the parasite. Immunofluorescence experiments showed that EaMIC3 binds only to specific sites located in chicken duodenal epithelial cells (25). This protein, as a critical virulence factor in parasites, not only mediates sporozoite invasion, but also inhibits host cell apoptosis. *Neospora caninum* can inhibit apoptosis through EGFR signal transduction activated by interaction between the functional domain of epidermal growth factor (EGF) in MIC3 protein and the EGF receptor of host cells (30, 31). Additionally, the MIC3 EGF domains in *T. gondii* inhibit cell apoptosis and promote parasite survival by activating the EGFR-AKT signaling pathway in host cells (21). In conclusion, this study suggests that *E. acervulina* merozoites may inhibit duodenal epithelial cell apoptosis by modulating MIC3 protein expression in the early stage of infection.

EaMIC3 has seven MAR domains but no functional EGF domain. These studies suggest that EaMIC3 inhibits apoptosis through specific receptors other than EGFR in duodenal epithelial cells. In previous studies, the E3 ubiquitin ligase CBL was obtained through a yeast cDNA library screening of chicken duodenal epithelial cells by yeast two-hybrid with *E. acervulina* MIC3 as the bait. The interaction between MIC3 and E3 ubiquitin ligase CBL was also confirmed by immunoprecipitation (23).

Ubiquitination is a posttranslational modification in eukaryotic cells. This process requires ubiquitin-activating enzyme E1, ubiquitin-binding enzyme E2, and ubiquitin ligase enzyme E3. E1 binds and activates ubiquitin and transfers it to E2. E3 recruits specific substrates and E2, and E2 then transfers the ubiquitin to the target proteins (32). In human cells, Fas and DR4/5 are members of the death receptor family. They can induce apoptosisby binding to specific death ligands. C-Cbl can degrade Fas and DR4/5 by ubiquitination and then inhibit cell apoptosis in T-lymphoma and prostate cancer. Interference of c-Cbl expression can promote apoptosis by upregulating the expression of Fas and DR4/5 (33, 34). Also, c-Cbl can degrade caspase-8 by ubiquitination and then inhibit cell apoptosis in gastric cancer (35). This result suggests that c-Cbl can inhibit host cell apoptosis *via* substrate protein ubiquitination.

In conclusion, the research has shown that chicken CBL has 82% homology with human c-Cbl. Overexpression of CBL inhibits duodenal epithelial cell apoptosis, while interference of CBL expression promotes apoptosis. This suggests that chicken CBL has a similar function as human c-Cbl in inhibiting the cell apoptosis pathway. Furthermore, this study confirmed that the number of apoptotic cells in the shRNA CBL+ MIC3 group was insignificantly less than that in the shRNA CBL group but significantly more than that in the MIC3 group. This result suggests that the inhibitory effect of *E. acervulina* MIC3 on duodenal epithelial cell apoptosis is related to CBL expression in the early stage. CBL may inhibit apoptosis by interacting with MIC3 or *via* its ubiquitination. The present study provides insights into *E. acervulina* infection and may aid prevention efforts and the control of coccidian parasites.

## Data Availability Statement

The original contributions presented in the study are included in the article/supplementary material, further inquiries can be directed to the corresponding author/s.

## Ethics Statement

The study was approved by the animal ethics committee of the College of Animal Science and Technology of Zhejiang A&F University.

## Author Contributions

CC, HS, and PW conceived and designed the experiments. PW, JL, YJ, YH, YZ, JX, SD, MC, and CG performed the experiments. PW, WW, CC, JW, and XZ analyzed the data. PW and CC wrote the paper. All authors contributed to the article and approved the submitted version.

## Conflict of Interest

The authors declare that the research was conducted in the absence of any commercial or financial relationships that could be construed as a potential conflict of interest.
